# Preparation and Properties of Foam Concrete Incorporating Fly Ash

**DOI:** 10.3390/ma15186287

**Published:** 2022-09-09

**Authors:** Dongsheng Zhang, Sen Ding, Ye Ma, Qiuning Yang

**Affiliations:** 1 School of Civil and Hydraulic Engineering, Ningxia University, Yinchuan 750021, China; 2 Research Group RecyCon, Department of Civil Engineering, KU Leuven, Campus Bruges, 8200 Bruges, Belgium

**Keywords:** foam concrete, foaming agent, fly ash, foam stabiliser, thermal conductivity, compressive strength, porosity

## Abstract

Foam concrete is fire resistant and durable and has broad applicability as a building insulation material. However, cement has high energy consumption and causes pollution, necessitating an environment-friendly cementitious material to replace the cement used to prepare foam concrete. In this study, foam concrete was prepared through chemical foaming. The influence of the foaming agent material, foam stabiliser, and fly ash on the basic properties of the foam concrete, including the dry bulk density, compressive strength, and thermal conductivity, was studied, and the pore structure was characterised. The results show that with an increase in the hydrogen peroxide (H_2_O_2_) content, the dry bulk density, compressive strength, and thermal conductivity of foam concrete decreases, whereas the pore diameter increases (0.495 to 0.746 mm). When the calcium stearate content is within 1.8%, the pore size tends to increase (0.547 to 0.631 mm). With increase in the fly ash content, the strength of foam concrete gradually decreases, and the dry bulk density first decreases and then increases. When the blending ratio of fly ash is 10–40%, the thermal conductivity gradually decreases; an extreme thermal conductivity of 0.0824 W/(m·K) appears at the blending ratio of 40%, and the dry bulk density is 336 kg/m^3^.

## 1. Introduction

With increased focus on global energy conservation and emission reduction, building energy conservation, as an important part of energy conservation and emission reduction, has attracted extensive attention of researchers globally [1,2]. Building energy consumption in China accounts for approximately 30% of the total energy consumption in an area. Existing buildings have problems such as lack of thermal insulation measures or poor thermal insulation performance. The energy consumption for heating and cooling accounts for approximately 55% of the building energy consumption [3]. Moreover, factors such as ageing and poor fire resistance cause fire accidents and seriously threaten the life and property of residents. Therefore, it is necessary to improve the thermal insulation performance of the building envelope and reduce its heating. Inorganic thermal insulation materials have excellent fire performance and the same longevity as buildings. Therefore, researchers have attempted to develop new inorganic thermal insulation materials for use in buildings [4,5].

Foam concrete is a typical inorganic wall thermal insulation material. It has many advantages, such as light weight, good environmental protection performance, good thermal insulation performance, high scope for industrialisation, and convenient construction [6,7]. However, at present, foam concrete is not widely used for wall insulation owing to inadequate strength and dry bulk density [8]. The foaming agent and foam stabiliser are the key constituents affecting the performance of foam concrete. They affect the air content, fluidity, and volume stability of the fresh slurry and ultimately affect the dry bulk density and strength of the hardened body [9,10,11]. In addition, cementitious materials aid the cementation of foam concrete and are the source of its strength. Presently, a part of the cement used in the preparation of foam concrete is replaced with fly ash. The addition of fly ash reduces the amount of cement required, thus reducing the manufacturing cost of the product [12]. In addition, fly ash can enhance the foam concrete performance owing to microaggregation, improved morphology, and pozzolanic effect [13,14,15]. Wen et al. [16] prepared foam concrete with dry bulk density of approximately 200 kg/m^3^, but the utilisation rate of fly ash was only 20%. Li et al. [17] studied the preparation of foam concrete with a large amount of fly ash; the amount of fly ash was as high as 60%, but the dry apparent density of the foam concrete products prepared by them was as high as 1000 kg/m^3^. Li [18] showed that at the early stage of hardening, when the water–binder ratio was constant, the strength of the foam concrete with a certain proportion of fly ash was low, and, as the proportion of fly ash increased, the strength gradually decreased. The results of Zhu et al. [19] showed that the strength of low-density foam concrete mixed with fly ash was lower than that of the control group without fly ash, and the degree of influence on the strength was related to the density. Qiu et al. [20,21] prepared foam concrete with a large amount of fly ash through a chemical method. The authors found that a test block with very low water-to-cement ratio was easily cracked, and the test block with very high water-to-cement ratio easily formed a through-hole structure, which affected the performance of the foam concrete. Jones et al. [22] studied the use of untreated low-lime fly ash as a substitute for sand in foam concrete, and the tested dry apparent density ranged from 1000 to 1400 kg/m^3^. The research shows that fly ash can obviously improve the strength of foam concrete and has stronger fluidity; however, it was found that there was a need to increase greatly the amount of foam required to achieve the specified design plastic density.

Generally, the thermal insulation performance of foam concrete can be improved by increasing the porosity [23]. Lian et al. [24] found that, similarly to several types of porous media, the strength of porous concrete is significantly affected by the porosity of its internal structure. Kearsley et al. [25] studied the influence of classified and unclassified fly ash, used to replace a considerable portion of cement, on the performance of foam concrete and proposed a relationship between porosity and compressive strength. The compressive strength of foam concrete is known to be a function of porosity and age, and the multiplication model is the most suitable model for the results of concrete age up to 1 year. Jiang et al. [26] produced a fire-resistant, low-cost, and highly porous foam concrete by adding preformed foam. The foamed concretes exhibited porosities in the range of 88.5–95.4%, compressive strengths in the range of 0.12–0.75 MPa, and low thermal conductivities in the range of 0.036–0.063 W/m·K.

It can be seen that the existing research on foam concrete has mostly focused on the influence of the composition on the performance, whereas the influence of pore structure has been neglected. The most remarkable feature of foam concrete structure is its high porosity, which makes it very different from ordinary concrete in performance. Therefore, it is of great significance to study the porosity of foam concrete for improving its performance. In this study, a foaming agent (H_2_O_2_) and foam stabiliser (calcium stearate) were used to prepare a cement-based fly ash foam concrete. According to the changes in the water–binder ratio and blending ratio of H_2_O_2_, calcium stearate, and fly ash, the influence of different parameters on the strength, dry bulk density, and thermal conductivity of foam concrete was studied. In addition, the influence of different parameters on the pore structure of foam concrete was studied through an image analysis method. The research results can provide technical support for the widespread application of fly ash foam concrete.

## 2. Materials and Methods

### 2.1. Materials

Ordinary Portland cement and fly ash were used as the cementing materials. The chemical compositions of cement and fly ash were detected using an X-ray fluorescence spectrometer (Primus-2, Japan), and the results are listed in Table 1. The physical properties of fly ash and cement are listed in Table 2. H_2_O_2_ with a concentration of 27.5% was used as the foaming agent; calcium stearate was used as the foam stabiliser, and lithium carbonate as the coagulant.

### 2.2. Design of Mix Ratio

Foam concrete was prepared using the chemical foaming method. The fixed water–binder ratio was 0.56. The foaming agent contents were 5.5%, 6%, 6.5%, 7%, and 7.5% (A1–A5); the corresponding amounts of foam stabiliser were 1.2%, 1.4%, 1.6%, 1.8%, and 2.0% (A2, B1–B4), respectively, and the replacement rates of fly ash were 0%, 10%, 20%, 30%, 40%, 50%, and 60% (B3, C1–C6), respectively. The foam concrete test blocks were prepared according to these proportions, and the physical and mechanical properties were tested. In order to prevent the specimen from collapsing and reduce the setting time of foam concrete, a certain amount of lithium carbonate was added to foam concrete based on the previous experimental research. Table 3 lists the proportions of the constituents.

### 2.3. Preparation and Curing

**Figure 1:**
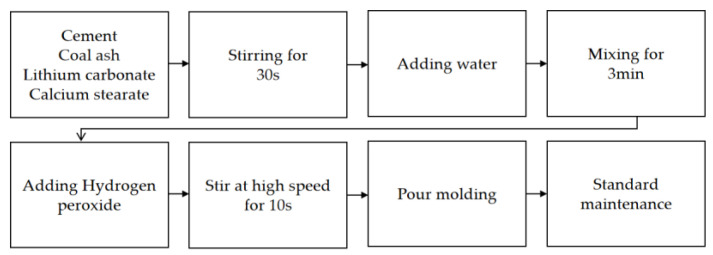
Preparation of foam concrete.

The preparation is illustrated by the flow chart in Figure 1. First, the cement, fly ash, calcium stearate, and other admixtures were weighed in a certain proportions and dry mix until uniform. Thereafter, the weighed water was poured into the mixer and mixed for 3 min. The slurry was stirred at high speed for another 10 s, and then quickly poured into the mould. After 6 h, the foam concrete was cut above the mould with a cutting knife. After standing for 1 d, the test piece was placed in the curing room for 28 d under standard curing conditions (at a temperature of 20 ± 2 °C and relative humidity of more than 95%).

### 2.4. Testing Methods

#### 2.4.1. Dry Bulk Density

Three indicators were tested according to the requirements in JG/T 266–2011 [27]. The size of the test sample was 100 mm×100 mm × 100 mm, and three samples were prepared for each composition. The dry bulk density was measured using the mass volume method. The axial dimensions of the test pieces were measured in the length, width, and height directions, accurate to 1 mm, and the volume V of the test piece was calculated. Then, the test piece was placed in an oven at (60 ± 5) °C for 24 h, followed by another 24 h at (80 ± 5) °C. The samples were then dried at (105 ± 5) °C to achieve a constant mass (M_0_). The drying process was repeated after an interval of 4 h, and the mass difference was not allowed to exceed 0.5% of the mass of the test piece. The dry bulk density was calculated as

(1) $$\rho =\frac{M_{0\;}}{V}\times 10^{6},$$

where ρ-dry bulk density, kg/m^3^; M_0_-mass of test piece after drying, g; V-volume of test piece, mm^3^.

#### 2.4.2. Thermal Conductivity

According to GB/T 10294-2008 [28], the size of the insulation board used in the test was 300 mm × 300 mm × 30 mm. The ADH3030 thermal conductivity tester was used for the testing. Before the test, two insulation boards which had reached the curing age were dried for 3 days at 105 °C, cooled to room temperature (20 °C), and thereafter the upper, middle, and lower thicknesses of the two boards were measured. The thickness was taken as the average value of three measurements. The sample was then placed in the clamp of the thermal conductivity tester. The thickness of the test piece was input as the data setting. The instrument automatically measured the results every 4 h, and the thermal conductivity was automatically tested after four times.

#### 2.4.3. Compressive Strength

According to Chinese JG/T 266–2011 [27], the cured specimen was placed in an electric oven maintained at (60 ± 5) °C for 24 h, dried at (80 ± 5) °C for 24 h, and subsequently dried at (105 ± 5) °C for 24 h until a constant weight was achieved. The sample was then removed, and an electro-hydraulic servo universal testing machine controlled by a WAW-2000 microcomputer was used to measure the compressive strength of the foam concrete. The average value of the tests was taken as the compressive strength of each test sample.

#### 2.4.4. Pore Structure Parameter Test

**Figure 2:**
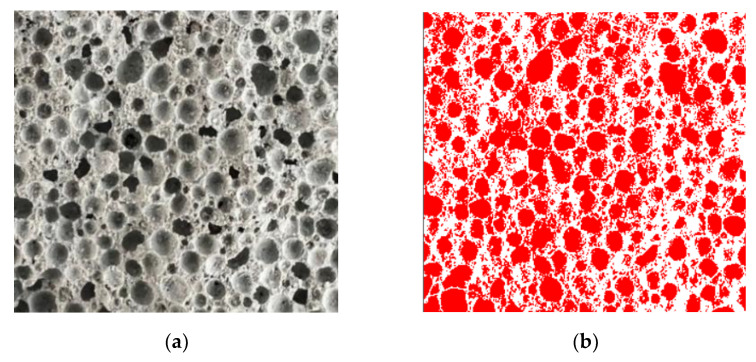
Typical initial image and processed image of the foamed concrete: (**a**) 10 mm × 10 mm section; (**b**) processed image 10 mm × 10 mm section.

The pore structure parameters of foam concrete were measured using the image analysis method. After 28 days of curing, the test piece was placed in an electric oven and heated at (80 ± 5) °C for 24 h. A cutting tool was used to cut along the direction parallel to the porous surface, and an abrasive paper was used to remove the powder particles on the surface. The sample was photographed using an AO-HD208CD electron microscope to obtain the image information, as shown in Figure 2a. The captured photos were then imported into Photoshop software, the pore structure data analysed through binary processing (the red part represents pores, Figure 2b), various test parameters built into Image Pro Plus software, and the data finally imported into the Origin software for analysis. Parameters such as porosity and pore size were obtained.

## 3. Results and Discussion

### 3.1. Dry Bulk Density

**Figure 3:**
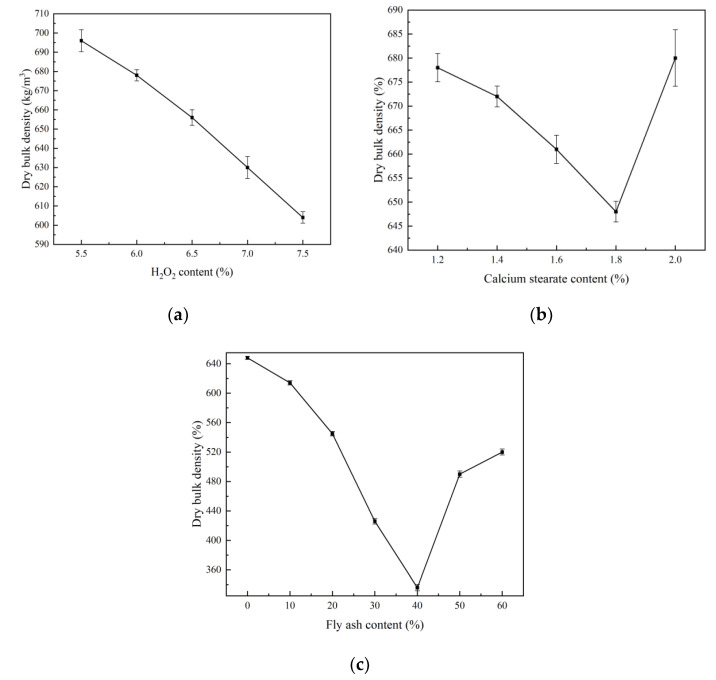
Dry bulk density of foamed concrete: (**a**) H_2_O_2_ content; (**b**) calcium stearate content; (**c**) fly ash content.

Foaming agent is one of the basic raw materials for preparing foam concrete. The foaming agent reacts chemically in the evenly mixed slurry to generate a large amount of gas, which is distributed in the slurry and gradually trapped in the hardened concrete with the solidification of the slurry, forming a uniform and stable porous structure [29,30]. Therefore, the density of foam concrete mainly depends on the amount of foaming agent added. It can be seen from Figure 3a that the dry bulk density of the test piece decreases with an increase in the amount of foaming agent. This is because the addition of the foaming agent increases the number of internal pores of the concrete, increases the proportion of the gas phase, and decreases the proportion of the solid phase [11]. Therefore, the dry bulk density of foam concrete decreases.

The addition of the foam stabiliser causes the dry bulk density of the foam concrete to decrease first and then increase (Figure 3b). When the calcium stearate content is 1.8%, the dry bulk density of the foam concrete is the lowest at 648 kg/m^3^, which is 4.42% lower than that of foam concrete with 1.2% H_2_O_2_. This is because the water in the formed pore structure will gradually decrease until it is completely consumed during the hydration process. The mechanical and elastic strengths of the original liquid film are improved under the action of calcium stearate, enabling the liquid film to better withstand the external forces without breaking. Therefore, the stability of the bubbles is improved, escaping of the bubbles is reduced, the number of pores per unit volume is increased, and dry bulk density of the foam concrete is decreased. However, when the calcium carbide content increases from 1.8% to 2.0%, the dry density of the foam concrete increases, which may be because of the consistency of the cement paste and the increase in bubble formation resistance. When the dissolution of calcium stearate reaches the limit, the excessive calcium stearate causes a delay in hydration to a certain extent, resulting in a decrease in the compressive strength and an increase in the dry bulk density [31].

With the increase in fly ash content, the dry bulk density of foam concrete shows a trend towards initial decrease and subsequent increase (Figure 3c). When the fly ash content is 40%, the density of foam concrete is 336 kg/m^3^, which is much lower than that foam concrete with 0–20% fly ash. This is because the density of fly ash is lower than that of cement. If fly ash is used to replace a part of the cement, the density of the foam concrete will naturally decrease. Secondly, fly ash can effectively reduce the friction resistance between the pastes as well as reduce the resistance to bubble formation during foaming. However, when the fly ash content reaches 50–60%, the dry bulk density of foam concrete increases slightly, which may be due to further reduction in the cement content of the system. Very high fly ash content makes the slurry flow rapidly, affecting the foaming effect, and the trapped gas escapes, thus increasing the dry bulk density [32].

### 3.2. Compressive Strength

**Figure 4:**
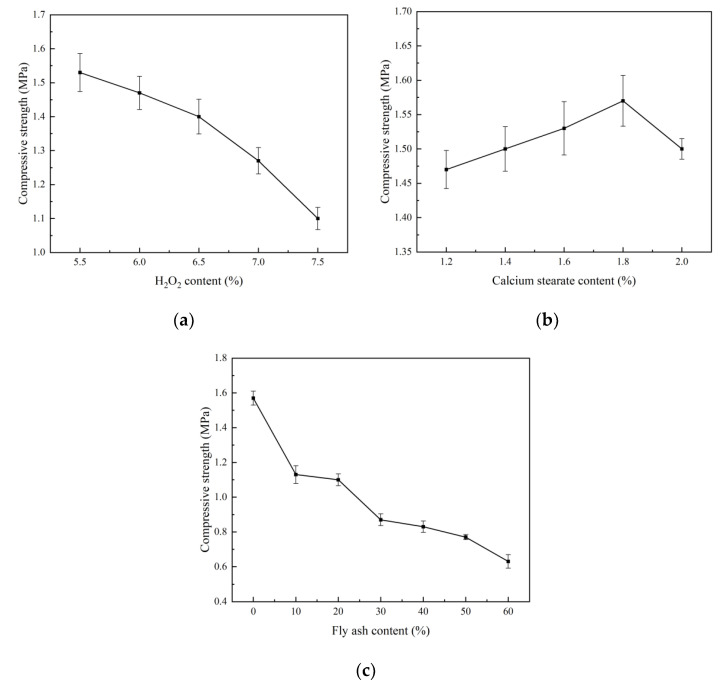
Compressive strength of foam concrete: (**a**) H_2_O_2_ content; (**b**) calcium stearate content; (**c**) fly ash content.

It can be seen from Figure 4a that the compressive strength of foam concrete gradually decreases with an increase in the foaming agent. When the amount of foaming agent is greater than 6.5%, the rate of decrease of the dry bulk density and strength increases noticeably. This is because when the amount of H_2_O_2_ is very large, the number of pores in the system is too high, the pore walls become thin, and the number of connected pores is relatively large, resulting in decreased strength [33,34]. The addition of calcium stearate causes the strength of the foam concrete to initially increase and then decrease (Figure 4b). When the amount of calcium stearate is 1.8%, the compressive strength reaches the maximum, which is 1.57 MPa; this represents an increase of 6.8% when compared with the amount of calcium stearate (1.2%). Regarding the physicochemical action, calcium stearate is insoluble in water and has good dispersibility and hydrophobicity. It allows bubbles to exist independently and reduces the probability of formation of connecting holes. As a foam stabiliser, it is evenly distributed in the liquid film of the bubbles in the slurry, which can reduce the liquid film discharge rate, prolong the bubble film rupture time, and improve the probability of pore formation. Therefore, the compressive strength of the foam concrete increases. When the calcium stearate is increased from 1.8% to 2.0%, the dissolution of calcium stearate reaches the limit, and excess calcium stearate powder accumulates at the pore wall. This is not conducive to the hydration reaction of the cement slurry, and a weak area is formed in the pore wall, resulting in the reduction of compressive strength [35].

The effect of fly ash on the strength of foam concrete is shown in Figure 4c. With an increase in the fly ash content, the compressive strength of the foam concrete shows a downward trend. Because fly ash can improve the fluidity of concrete, an increase in the fly ash content from 10% to 20% results in an increase in the number of bubbles per unit volume in the system, which will lead to a decrease in dry density, thinning of the bubble wall, and reduced compressive strength. However, the reduction is not evident, which makes the pore diameter more uniform and improves the pore structure. When the fly ash content increases from 20% to 30%, the compressive strength decreases rapidly. This is because the fluidity of the foam concrete slurry is enhanced with the increase in fly ash content; the expansion effect of the slurry becomes more obvious; the number of bubbles per unit volume in the system increases, and pore wall thickness decreases. The bearing capacity of the foam concrete decreases, thus reducing the compressive strength. Conversely, with increase in the fly ash content, the proportion of cement in the foam concrete system decreases; thus, the formation of gel hydrate of the cement reduces and the strength decreases. However, when the fly ash content increases from 30% to 60%, the compressive strength decreases more slowly, which may be due to the pozzolanic effect of fly ash, that is, the Al_2_O_3_ and SiO_2_ siliceous materials in fly ash and cement are mixed with water to generate alkaline activators such as Ca(OH)_2_ and further generate hydrated calcium silicate gel, which also contributes to the strength of the foam concrete cement stone [36,37,38].

### 3.3. Thermal Conductivity

Thermal conductivity characterises the thermal insulation performance of the material [39]. Foam concrete consists of two phases, i.e., solid phase and gas phase. The defects in the solid phase are not conducive to the reduction of the thermal conductivity, and the dominant factor for reducing the thermal conductivity is the gas phase. Therefore, the smaller the pore defects, the better the insulation effect of the porous material with less connectivity.

**Figure 5:**
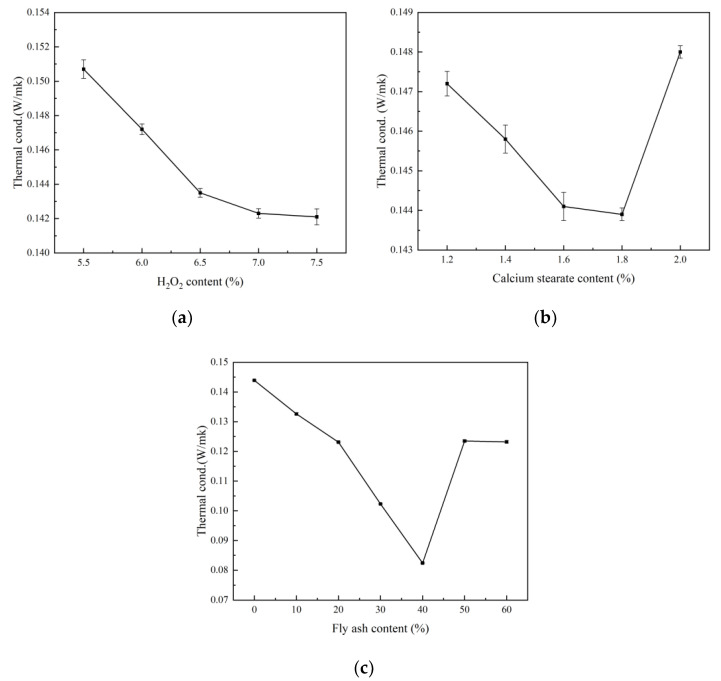
Compressive strength of foam concrete: (**a**) H_2_O_2_ content; (**b**) calcium stearate content; (**c**) fly ash content.

It can be seen from Figure 5a that in the foam concrete, as the amount of H_2_O_2_ increases (5.5~7.5%), the thermal conductivity decreases. The thermal conductivity values of foam concrete with 5.5%, 6%, 6.5%, 7%, and 7.5% H_2_O_2_ content were 0.1507, 0.1472, 0.1435, 0.1423, and 0.1421 W/(m·K), respectively. With the addition of the foaming agent, the porosity of the foam concrete increases and dry bulk density decreases. However, the high porosity is not conducive to heat conduction. Therefore, thermal conductivity shows a downward trend.

The thermal conductivity of the foam concrete decreases first and then increases with increase in the calcium stearate content, as shown in Figure 5b. The thermal conductivity values of foam concrete with 1.2%, 1.4%, 1.6%, 1.8%, and 2.0% calcium stearate were 0.1472, 0.1458, 0.1441, 0.1439, and 0.1480 W/(m·K), respectively. When the calcium stearate content was 2.0%, the excess calcium stearate increased the dry bulk density of the foam concrete and the thermal conductivity.

It can be seen from Figure 5c that when the proportion of fly ash in the foam concrete is 10–40%, the thermal conductivity decreases significantly. The pore wall in the hardened body is further compacted by the hydrated calcium silicate gel generated by the secondary hydration reaction of fly ash, and the number of pores increases, which greatly reduces the heat transfer efficiency. However, the specific surface area of the fly ash particles is high, which also hinders the heat transfer to a certain extent, causing the thermal conductivity to decrease significantly. When the fly ash content is increased from 40% to 60%, the excessive fly ash content increases the fluidity of the slurry, which allows the trapped gas to escape, decreases the number of pores, and increases the dry bulk density and thermal conductivity.

From the comprehensive dry bulk density and thermal conductivity results, when the content of fly ash is 40%, foam concrete has the minimum dry bulk density (336 kg/m^3^) and the minimum thermal conductivity (0.0824 W/(m·K)), and its strength is 0.63 MPa. Although it does not have advantages in strength compared with conventional concrete and geopolymer concrete, foam concrete is a lightweight thermal insulation material. In this paper, river sand is not added to the preparation of foam concrete, which makes it not usable in load-bearing structures but can be used in roof insulation, subgrade backfill, and other projects, meeting relevant standards.

### 3.4. Pore Structure Analysis

**Figure 6:**
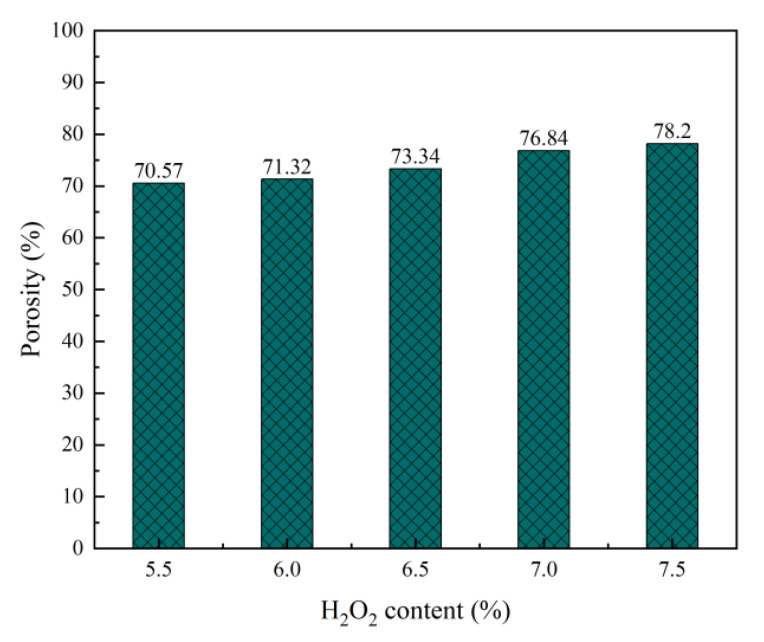
Effect of H_2_O_2_ content on porosity of foam concrete.

**Figure 7:**
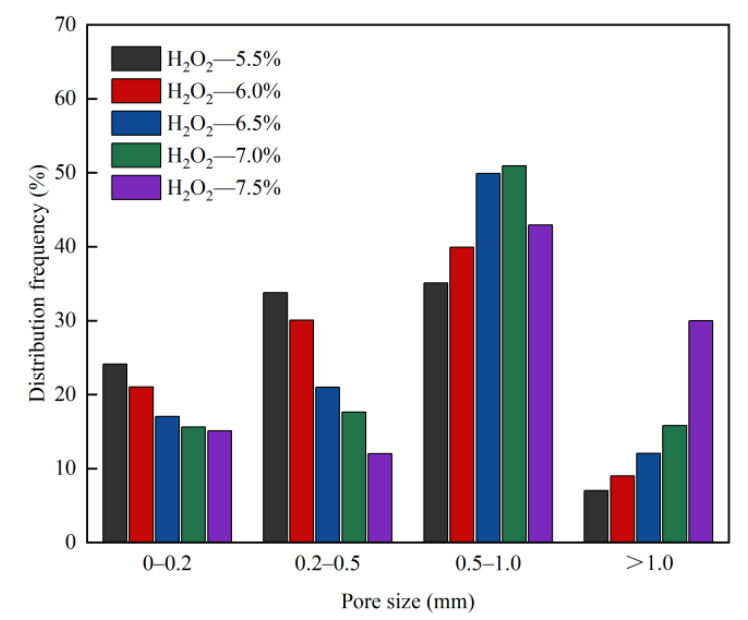
Effect of H_2_O_2_ content on pore size distribution frequency of foam concrete.

**Figure 8:**
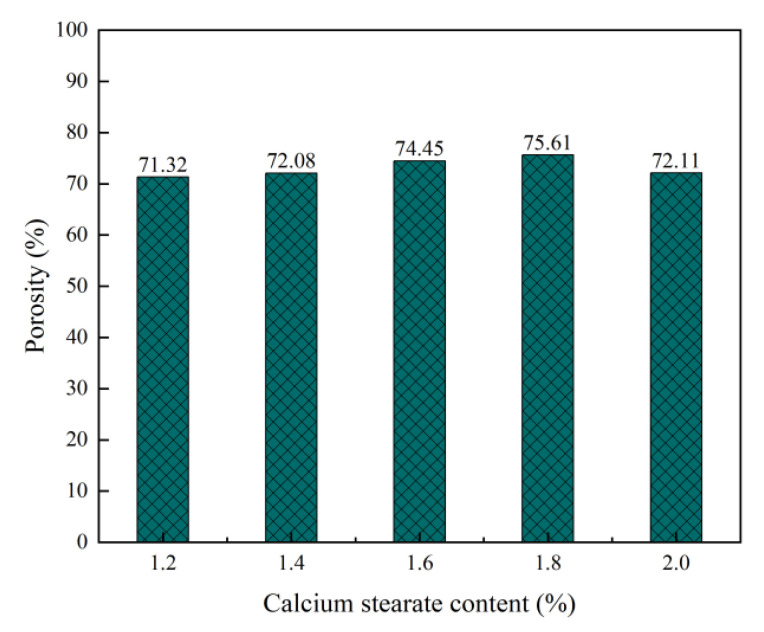
Effect of calcium stearate content on porosity of foam concrete.

**Figure 9:**
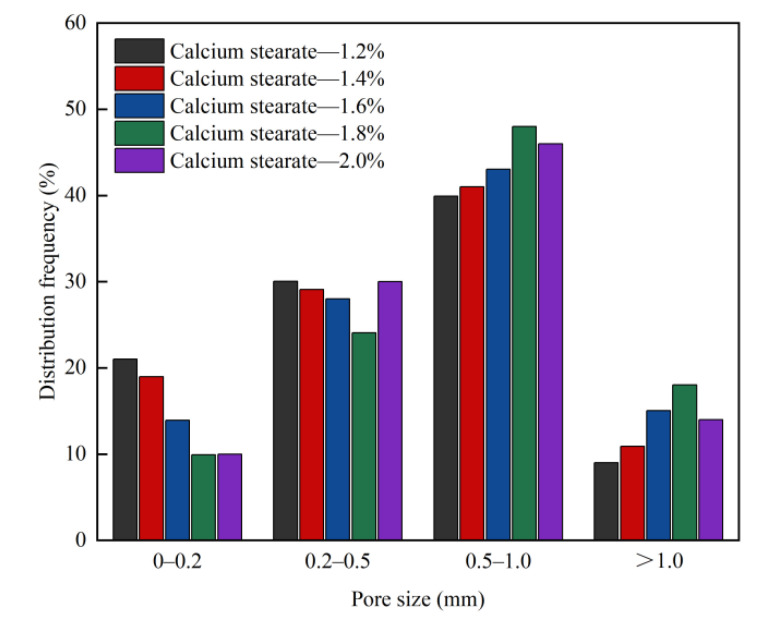
Effect of calcium stearate content on pore size distribution frequency of foam concrete.

**Figure 10:**
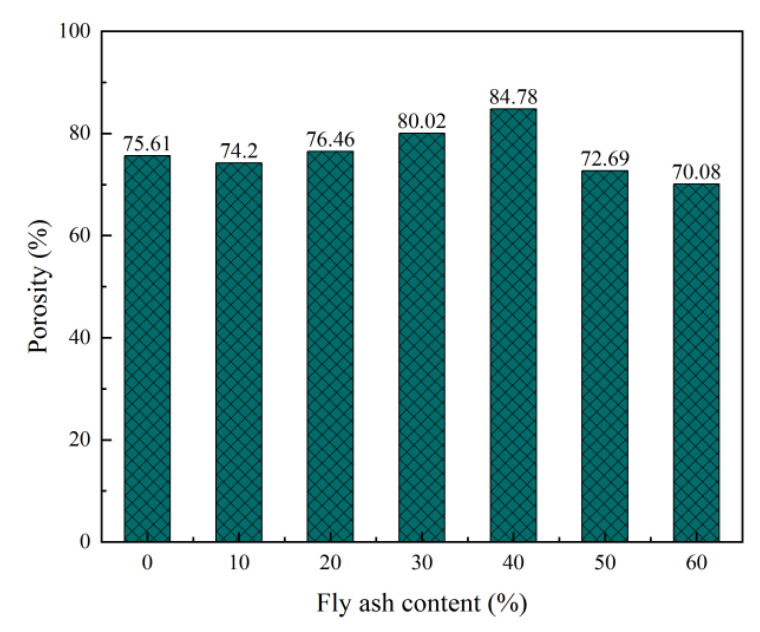
Effect of fly ash content on the porosity of foam concrete.

**Figure 11:**
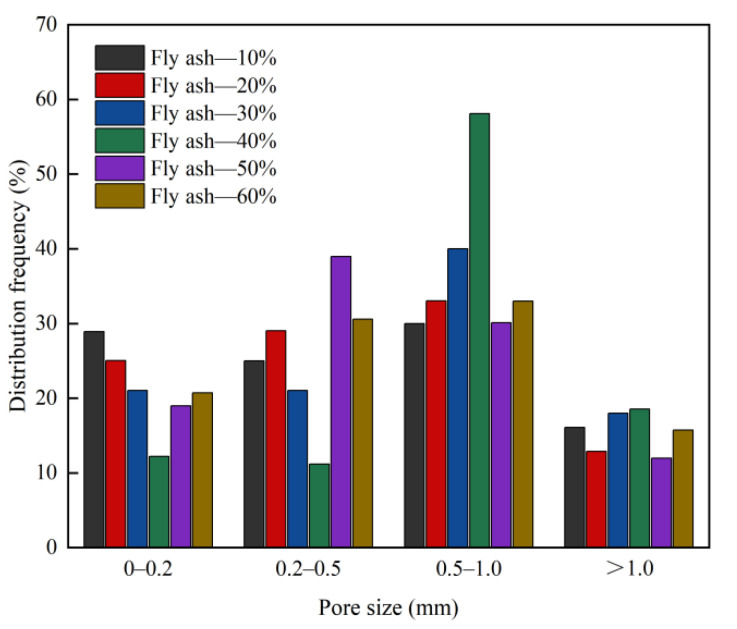
Effect of fly ash content on pore size distribution frequency of foam concrete.

The pore structure is an important factor affecting the strength, shrinkage, and durability of foam concrete, whereas the porosity, pore size distribution, dry bulk density, compressive strength, and thermal conductivity of foam concrete are closely related [40,41]. The influence of the contents of the foaming agent, foam stabiliser, and fly ash on the pore structure parameters of the foam concrete is shown in Figure 6, Figure 7, Figure 8, Figure 9, Figure 10 and Figure 11.

In the foam concrete system, the foaming agent is the key material for pore formation. After high-speed mixing, the foaming agent is evenly dispersed in the slurry. The bubbles in the foam concrete system gradually set and harden with the solidification of the slurry and finally form a porous structure. It can be seen from Figure 6 and Figure 7 that with increase in the H_2_O_2_ content (5.5–7.5%), the number of bubbles introduced in the foam concrete system increases, porosity gradually increases, fusion probability between the bubbles increases, probability of small aperture bubbles merging into large aperture bubbles increases, and dry bulk density gradually decreases [42]. When the porosity increases, the specific surface area also increases, resulting in reduction in heat transfer efficiency. Therefore, the thermal conductivity of the foam concrete decreases with an increase in porosity. In addition, it can be seen that the proportions of pores with pore diameters of less than 0.2 mm and those with pore diameters of 0.2–0.5 mm decrease; the proportion of pores with pore diameters of more than 1 mm increases; the number of pores increases; the pore walls of the pores become thinner, and the bearing capacity decreases. This also explains why the strength of the foam concrete decreases with increase in the H_2_O_2_ content from the perspective of the pore structure.

It can be seen from Figure 8 and Figure 9 that as the mixing proportion of the foam stabiliser increases, the porosity of the foam concrete increases first and then decreases. This is because the porosity of foam concrete mainly depends on its dry density. As the dry density of foam concrete decreases, its porosity gradually increases. The retention and accumulation of gas is key to the formation of bubbles. Bubble damage is mainly caused by low mechanical strength, short life, and poor stability of the liquid film. When the blending ratio of the foam stabiliser is low (1.2%), the proportion of pores with pore diameters less than 0.2 mm decreases, the proportion with pore diameters within 0.5–1.0 mm increases, and the proportion with pore diameters exceeding 1 mm increases. In addition, the content of calcium stearate is low, surface tension of the liquid film is large, mechanical strength of the bubble is low, and water loss is low. Hence, the bubbles are damaged, and because of this, the probability of large bubbles is reduced, the proportion of small bubbles is high, and the proportions of pores with pore sizes of less than 0.2 mm and 0.2–0.5 mm are high; consequently, the conversion of the liquid film to pores is biased towards small pores. When the blending ratio of calcium stearate is increased (1.2–1.8%), the surface tension of the bubble liquid film is reduced, the mechanical strength of the bubbles is increased, and time for the liquid film to break is delayed [43], giving the gel layer more time to replace the liquid film. The proportion of pores with pore diameters of 0.5–1 mm is increased, and conversion of the liquid film to pores tends to be widespread. However, the calcium stearate reduces the probability of bubble fusion, increases the number of closed pores, and reduces the stress concentration caused by connected pores. When the content of foam stabiliser is increased from 1.6% and 1.8%, the porosity remains similar, while the pore size and thickness increases. Therefore, the content of bubbles in the test block increases, and the bubbles are not easily destroyed, resulting in an increase in porosity. When the content of the foam stabiliser is 2%, the proportion of pores with pore sizes in the range of 0.2–0.5 mm increases, and that of the pores with sizes in the range of 0.5–1 mm decreases. This reduces the porosity and leads to a decrease in the porosity of the foam concrete.

Adding fly ash as a mineral admixture in foam concrete can help in achieving more uniform pore distribution [13,44]. Fly ash has fine particle sizes. By adsorbing on each bubble, it provides an effective and uniform coating to prevent the bubble from merging and overlapping, improves the pore structure of the foam concrete and densifies it, and further reduces the dry bulk density and thermal conductivity of the foam concrete. Figure 10 and Figure 11 indicate that an increase in the fly ash content causes the porosity of the foam concrete to first increase and then decrease. When the content is 40%, the porosity reaches the peak value of 84.78%. Regarding pore size distribution, as the proportion of fly ash increases (10–40%), the proportion of pores with pore sizes lesser than 0.2 mm decreases, the proportion of pores with pore sizes of 0.5–1.0 mm increases, and the proportion of pores with pore sizes greater than 1 mm increases. When the proportion of fly ash continuously increases (50–60%), the proportion of holes with pore diameters of 0.2–0.5 mm decreases, whereas the proportion of holes with pore diameters of 0.5–1 mm increases. When the proportion of fly ash is in the range of 10–20%, its morphological effect improves the pore structure of foam concrete [45,46], and the proportion of pores larger than 1 mm decreases. When the mixing ratio of fly ash is 10–40%, the liquid film adheres to a large number of cement particles due to the adsorption of the surfactant. The fly ash particles are fine and easily absorbed by the liquid film. These solid particles play the role of reinforcing the bubbles. Therefore, the number of pores in the foam concrete and its porosity increase, whereas the dry bulk density, strength, and thermal conductivity decrease. Excessive proportion of fly ash (50–60%) will not enhance the performance of foam concrete. This is because the cementitious material content in the matrix of the foam concrete decreases, and a large amount of fly ash is not conducive to the early hydration of the foam concrete. When the proportion of fly ash is too large, the amount of cementitious materials decreases, and the total amount of hydration products and total amount of hydration heat are greatly reduced. This is not conducive to the thickening and setting of the slurry and worsens the gelling property of the foam concrete [47]. The number of pores as well as the pore size is reduced, resulting in a certain increase in the dry bulk density and decrease in strength.

## 4. Conclusions

The effects of different contents of H_2_O_2_, calcium stearate, and fly ash on the strength, dry bulk density, thermal conductivity, and pore structure of foam concrete were studied. A foam concrete insulation material capable of effectively utilising the solid waste fly ash and delivering the basic performance requirements was prepared.

(1) The dry bulk density, strength, and thermal conductivity of foam concrete decrease with the increase in foaming agent.
(2) With the addition of foam stabilizer, the dry bulk density and thermal conductivity of foam concrete decrease first and then increase, while the compressive strength increases first and then decreases. When the optimum content of foam stabilizer is 1.8%, the dry bulk density of foam concrete is 648 kg/m^3^, the compressive strength is 1.57 MPa, and the thermal conductivity is 0.1439 W/(m·K).
(3) The dry bulk density and thermal conductivity of foam concrete decrease first and then increase with the increase of fly ash, while the compressive strength decreases gradually. When the content of fly ash is 40%, foam concrete has the minimum dry bulk density (336 kg/m^3^) and the minimum thermal conductivity (0.0824 W/(m·K)), and the strength is 0.63 MPa.
(4) With the increase of H_2_O_2_ content (5.5~7.5%), the porosity gradually increases, and the pore size tends to increase. The appropriate content can improve the pore structure of foam concrete. When the content of foam stabilizer is less than 1.8%, the pore size tends to increase. A small amount of fly ash (10–20%) has the morphological effect of improving the pore structure of foam concrete and making the pore size smaller. When the content of fly ash is 40%, the performance is the best, and the porosity is the highest. The pore size distribution is more uniform, which makes it possible to control the pore structure, significantly reduce the dry bulk density of foam concrete, and significantly reduce the thermal conductivity.

In this paper, the strength of foam concrete made of fly ash is low. In order to improve foam concrete for application in practical projects, the strength of fly ash foam concrete can be improved by alkali excitation technology, and fibre can be added to improve its crack resistance in future research. In addition, the influence of the materials constituting foam concrete on durability performance, especially frost resistance, also needs to be studied in the future.

## Figures and Tables

**Table 1:** Chemical properties of cement and fly ash (%).

Oxide	Al_2_O_3_	SiO_2_	Fe_2_O_3_	CaO	Na_2_O	K_2_O	P_2_O_5_	SO_3_
Cement	7.60	22.46	5.00	57.15	0.31	0.86	0.105	2.96
Fly ash	26.38	47.85	8.43	5.81	1.11	2.22	1.17	1.94

**Table 2:** Physical composition of cement and fly ash.

Properties	Cement	Fly Ash
Fineness (% retained on 45 μm sieve)	7.60	17.4
Water demanded (%)	-	97.0
Loss on ignition (%)	1.4	5.84

**Table 3:** Proportions of different mixtures (kg/m^3^).

Code	Water	H_2_O_2_	Calcium Stearate	Fly Ash	Cement	Lithium Carbonate
**A1**	224	22	4.8	0	400	1.36
**A** **2**	224	24	4.8	0	400	1.36
**A** **3**	224	26	4.8	0	400	1.36
**A** **4**	224	28	4.8	0	400	1.36
**A** **5**	224	30	4.8	0	400	1.36
**B1**	224	24	5.6	0	400	1.36
**B** **2**	224	24	6.4	0	400	1.36
**B** **3**	224	24	7.2	0	400	1.36
**B** **4**	224	24	8.0	0	400	1.36
**C1**	224	24	7.2	40	360	1.36
**C** **2**	224	24	7.2	80	320	1.36
**C** **3**	224	24	7.2	120	280	1.36
**C** **4**	224	24	7.2	160	240	1.36
**C** **5**	224	24	7.2	200	200	1.36
**C** **6**	224	24	7.2	240	160	1.36

## Data Availability

Not applicable.
